# A comparative study of online and face-to-face gatekeeper training using the suicide CARE program

**DOI:** 10.3389/fpsyt.2025.1682318

**Published:** 2025-10-15

**Authors:** Ah Rah Lee, Sung Moon Choi, Hwa-Young Lee, Soung Nam Kim, Jeungsuk Lim, Sang Min Lee, Jong-Woo Paik

**Affiliations:** ^1^ Department of Psychiatry, Kyung Hee University College of Medicine, Kyung Hee University Hospital, Seoul, Republic of Korea; ^2^ Department of Psychiatry, College of Medicine, Soonchunhyang University Seoul Hospital, Soonchunhyang University, Seoul, Republic of Korea; ^3^ Dongdaemun-Gu, Primary Mental Health Welfare Center, Seoul, Republic of Korea; ^4^ Happy Care Together, Seoul, Republic of Korea

**Keywords:** suicide prevention, gatekeeper training, suicide CARE, online education, face-to-face education, community mental health

## Abstract

**Background:**

Gatekeeper training programs are essential public health strategies for suicide prevention. With the rapid digitization of health education, evaluating the effectiveness of online gatekeeper training relative to traditional face-to-face training has become increasingly important.

**Objectives:**

This study compared the effectiveness of online and face-to-face formats of the standardized Suicide CARE 2.0 gatekeeper training in enhancing suicide prevention knowledge, attitudes, behaviors, and preparedness among community mental health workers in South Korea. We tested the non-inferiority of the online format in improving key outcomes.

**Methods:**

A quasi-experimental, two-group pre–post design was employed with 99 participants (51 face-to-face, 48 online) recruited from community mental health centers. Participants were randomly assigned to either the online or face-to-face gatekeeper training group using a computerized randomization tool (www.randomizer.org). Both groups received identical content delivered by the same instructor. Outcomes assessed included self-perceived knowledge, factual knowledge, preparedness to help, attitudes toward suicide, and suicide prevention behaviors. Analyses included paired t-tests and ANCOVA, with effect sizes (Cohen’s d, partial η²) and 95% confidence intervals reported.

**Results:**

Both groups significantly improved all five domains. The online group showed greater improvements in self-perceived knowledge, preparedness, and behaviors (p < 0.001), while the face-to-face group demonstrated larger gains in factual knowledge (p = 0.017). Effect sizes supported the practical relevance of these findings. Both groups exhibited positive shifts in attitudes, with the online group showing more pronounced changes in avoidant attitudes and readiness to intervene. However, changes in deeply entrenched beliefs, such as the normalization of suicide, were limited.

**Conclusion:**

Online gatekeeper training is a feasible and effective alternative to face-to-face instruction, particularly in settings with limited resources or during emergencies. While each format offers distinct advantages, hybrid models may yield the most comprehensive benefits. These findings support the inclusion of scalable online training in national suicide prevention strategies. Suicide prevention, gatekeeper training, Suicide CARE, online education, face-to-face education, and community mental health.

## 1 Introduction

Suicide remains one of the most pressing public health concerns worldwide. South Korea continues to report the highest suicide rate among OECD countries, emphasizing the urgency of culturally relevant suicide prevention strategies (1). In response, gatekeeper training programs have been developed to train non-clinicians, such as educators, community workers, and family members, with the skills needed to recognize warning signs and refer high-risk individuals to professional help. In South Korea, the standardized gatekeeper training program known as “Suicide CARE (Careful Observation, Active Listening, Risk Evaluation and Expert Referral)” was introduced in 2011 to address cultural tendencies toward emotional suppression and to promote early suicide risk detection (2). The program has since trained over five million individuals and has evolved into multiple versions targeting specific groups such as adolescents, soldiers, teachers, and firefighters (2–4).

With the increasing integration of digital technologies into public health education, numerous studies have examined whether online gatekeeper training can achieve outcomes comparable to traditional face-to-face formats (5–7). While evidence suggests that both modalities are effective in improving suicide-related knowledge, attitudes, and self-efficacy (6), there remain concerns regarding the quality of learner engagement, long-term knowledge retention, and the affective depth of learning, especially in online formats (7). These concerns are particularly relevant because online programs often lack interpersonal interaction, emotional resonance, and experiential learning activities, like role-playing or group discussions, which are considered critical in suicide prevention training (7, 8). Furthermore, even in countries with mandated suicide prevention education, insufficient funding and poor quality control have led to superficial, checklist-based implementation, undermining the impact of such programs (9).

Against this background, this study evaluates whether an online delivery of the Suicide CARE program produces non-inferior outcomes when compared to face-to-face education. The core hypothesis is that, when the training content and instructional quality are held constant, online gatekeeper education can lead to equivalent gains in suicide prevention knowledge, attitudes, and behaviors. Establishing non-inferiority would support the scalability of online formats, particularly in resource-limited contexts or during public health emergencies, such as the COVID-19 pandemic.This study aimed to test whether online training is non-inferior to face-to-face training in improving suicide prevention knowledge, self-efficacy, and attitudes among community mental health workers.

## 2 Methods

### 2.1 Participants

Between July and December 2020, mental health professionals were recruited from community mental health centers and online platforms. Eligibility required current employment in the field and no gatekeeper training within the past year. Of 109 initial respondents, 99 completed both pre- and post-training assessments (face-to-face: n = 51; online: n = 48) and were included in the analysis. Participants were randomly assigned to either the online or face-to-face gatekeeper training group using a computerized randomization tool (www.randomizer.org). Baseline demographics (e.g., gender, age, education, religion, employment status, perceived economic status) were collected; only gender differed significantly between groups (p = 0.007). An *a priori* power analysis using G*Power 3.1, based on a medium effect size (Cohen’s d ≈ 0.50), indicated a minimum of 34 participants per group (α = 0.05, power = 0.80). The final sample exceeded this threshold, ensuring sufficient statistical power. All procedures adhered to the Declaration of Helsinki.

### 2.2 Study design and procedure

This study employed a single-session, pre-post design to compare the effectiveness of face-to-face versus online gatekeeper training using Suicide CARE Version 2.0, South Korea’s national suicide prevention curriculum (3, 4). Participants in both groups completed pre-training assessments (30 minutes), received a standardized 60-minute training session, and then completed post-training assessments (30 minutes). All training was conducted by the same certified instructor to control for instructor effects.

### 2.3 Intervention: suicide CARE version 2.0

The intervention was based on Suicide CARE Version 2.0, an evidence-based update of the national standard program (3, 4). This version includes three core modules: “Careful Observation,” which focuses on recognizing behavioral and verbal warning signs; “Active Listening,” which promotes empathetic dialogue with individuals at risk; and “Risk Evaluation and Expert Referral,” which teaches how to assess suicide risk and refer individuals to appropriate mental health services. The same content and instructor were used across both formats to ensure internal validity.

### 2.4 Measures

We assessed suicide prevention-related knowledge, attitudes, and behavioral intentions using a combination of validated and adapted instruments. Cronbach’s α values were calculated using the current study sample to assess internal consistency; the assessment tool comprised five subscales derived from validated instruments. Assessments were conducted immediately before and after the training session using structured self-report questionnaires. The Institutional Review Board approved the study protocol.

#### 2.4.1 Self-perceived suicide prevention knowledge

Participants’ subjective understanding of suicide risk factors and intervention strategies was assessed using a 9-item scale adapted from Wyman et al. (10) and translated into Korean by Ryu (11). Items were rated on a 7-point Likert scale (1 = “Not at all” to 7 = “Very much”), with higher scores indicating greater self-perceived knowledge. This scale has demonstrated strong internal consistency and cross-cultural applicability. In the current study, Cronbach’s α was 0.88, indicating high reliability.

#### 2.4.2 Factual knowledge about suicide

Objective suicide prevention knowledge was measured using 10 multiple-choice questions derived from the standardized “See, Listen, Speak” framework of the Korean Ministry of Health and Welfare (2). Each item was scored dichotomously (0 = incorrect, 1 = correct), with total scores ranging from 0 to 10. Higher scores reflected greater factual knowledge. The internal consistency of this scale in the current study was high (Cronbach’s α = 0.89).

#### 2.4.3 Preparedness to help

Participants’ perceived readiness to assist individuals at risk of suicide was measured using a 4-item scale developed by Baber and Bean and translated by Kim H (12). Items were rated on a 5-point Likert scale (1 = “Strongly disagree” to 5 = “Strongly agree”), with higher scores reflecting greater preparedness. The scale demonstrated excellent reliability in this study (Cronbach’s α = 0.91).

#### 2.4.4 Attitudes toward suicide

Attitudes were assessed using the Attitudes Toward Suicide Scale (ATTS), initially developed by Renberg and Jacobsson (13) and culturally adapted for Korea. The Korean version comprises 37 items across 10 subdomains (e.g., tabooing, preventability, normalization, autonomy), rated on a 5-point Likert scale. Higher scores in different subdomains indicate either stronger preventive attitudes or more permissive views, depending on item framing. In the present sample, subdomain reliabilities were acceptable.

#### 2.4.5 Suicide prevention behaviors

Gatekeeper behavioral intentions were assessed using an 8-item measure developed by Kim J (14). based on the framework by Wyman et al. (10). Items reflect the likelihood of performing specific behaviors such as asking about suicide or referring someone to professional help. Responses were recorded on a 5-point Likert scale (1 = “Very unlikely” to 5 = “Very likely”). The scale showed strong internal consistency in this study (Cronbach’s α = 0.87).

### 2.5 Statistical analysis

Data were analyzed using IBM SPSS Statistics 26.0. Paired t-tests were used to evaluate within-group pre-post changes. Between-group differences were tested using ANCOVA, controlling for baseline scores. Effect sizes were reported as Cohen’s d (within-group) and partial η² (between-group), along with 95% confidence intervals. Statistical significance was set at p <.05.

## 3 Results

### 3.1 Participant characteristics

Of the 99 participants included in the final analysis, 51 were allocated to the face-to-face training group (51.5%) and 48 to the online group (48.5%). A significant gender imbalance was noted, with a higher proportion of females in the face-to-face group and males in the online group (χ² = 7.279, p = 0.007). No significant differences were observed between groups in age, education level, employment status, religious affiliation, or perceived economic status (**Table 1**).

**Table 1:** Characteristics of the online and face-to-face training groups.

Variable		Total (n=99)		Online training		Face-to-Face training		P-value
		n	%	n	%	n	%	
Sex	Male	44	44.4	28	58.3	16	31.4	0.0070
	Female	55	55.6	20	41.7	35	68.6	
Age	Under 40 years	47	47.5	19	39.6	28	54.9	0.1272
	40 years or older	52	52.5	29	60.4	23	45.1	
Educational Attainment	High School or Less	35	35.4	15	35.4	10	19.6	0.1827
	College or Higher	74	64.6	33	64.6	41	80.4	
Religious Affiliation	None	53	53.5	27	56.3	26	51.0	0.5993
	Yes	46	46.5	21	43.8	25	49.0	
Employment Type	Full-time	65	66.6	31	64.6	34	66.7	0.3840
	Part-time	9	9.1	3	6.3	6	11.8	
	Self-employed / Employer	6	6.1	5	10.4	1	2.0	
	Unpaid Family Worker	3	3.0	1	2.1	2	3.9	
	Unemployed	15	15.1	8	16.7	7	13.7	
Economic Status	Lower-middle or below	26	26.3	14	29.2	12	23.5	0.6005
	Middle	58	58.6	26	54.2	32	62.7	
	Upper-middle or above	14	14.1	8	16.7	6	11.8	

Categorical variables are presented by n, %.

P value: Chi-square test between two groups.

#### 3.2.1 Knowledge and preparedness

Both training modalities led to statistically significant improvements in all key domains: self-perceived knowledge, objective factual knowledge, preparedness to help, and behavioral intention (p < 0.001 for all measures). Effect sizes were medium to large (Cohen’s d = 0.58–0.94), indicating meaningful psychological and educational change.

The online group demonstrated greater gains in self-perceived knowledge (d = 0.74, 95% CI [0.47, 1.01]) and preparedness (d = 0.73, 95% CI [0.46, 0.99]), with significant group × time interactions (partial η² = 0.09 and 0.11, respectively), suggesting enhanced self-efficacy and confidence to intervene. These outcomes are practically substantial, as they reflect readiness to apply learned skills in real-world scenarios, an essential goal of gatekeeper training.

**Figure 1:**
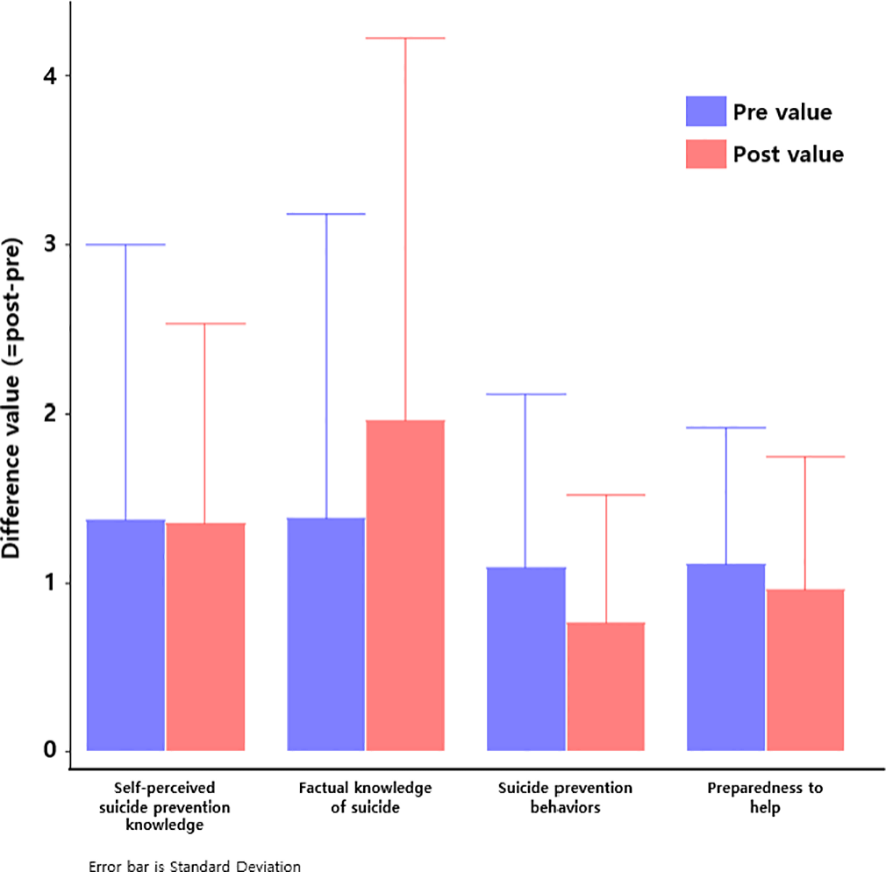
Pre-post differences in suicide prevention competencies across four domains. Bar graph displays mean changes from baseline to post-intervention in (1) self-perceived suicide prevention knowledge, (2) factual knowledge of suicide, (3) suicide prevention behaviors (behavioral intention), and (4) preparedness to help. Blue bars represent pre-intervention values; red bars represent post-intervention values. Error bars indicate standard deviations. All domains showed significant improvements from pre- to post- intervention (p<0.001), with the largest gains observed in factual knowledge and self-perceived preparedness.

Conversely, the face-to-face group showed greater gains in objective knowledge (d = 0.94, 95% CI [0.68, 1.19]; partial η² = 0.07), suggesting that in-person formats may be more effective for delivering dense factual or technical content. These findings underscore the strengths of each modality, depending on the intended learning objectives (**Table 2**, **Figure 1**).

**Table 2:** Pre–post changes in self-assessed knowledge, objective knowledge, help preparedness, and prevention behaviors.

Variable	Group	Pre	Post	Difference (=post-pre)†	p-value of group††
Self-perceived suicide prevention knowledge	Online	2.68±1.54	4.05±1.36	1.37±1.63^**^	<.0001
	Face-to-Face	3.73±1.27	5.08±1.04	1.35±1.18^**^	
Knowledge of suicide	Online	5.35±1.86	6.73±1.43	1.38±1.80^***^	0.0172
	Face-to-Face	4.24±1.88	6.20±2.12	1.96±2.26^***^	
Suicide prevention behaviors	Online	1.99±1.00	3.08±1.03	1.09±1.02^***^	<.0001
	Face-to-Face	3.10±0.95	3.86±0.67	0.76±0.76^***^	
Preparedness to help	Online	2.16±0.93	3.27±0.92	1.11±0.81^***^	<.0001
	Face-to-Face	2.95±0.80	3.91±0.55	0.96±0.79^***^	

A numerical variable is presented by mean±SD.

**†**Paired t-test between pre and post; *: <0.05, **: <0.01, ***: <0.001.

**††**P-value derived from ANCOVA, adjusted for baseline scores.

#### 3.2.2 Gatekeeper behavior

Both groups showed significant increases in their intention to engage in suicide prevention behaviors (p < 0.001). The online group exhibited a significantly larger change compared to the face-to-face group (d = 0.81, 95% CI [0.53, 1.09]; partial η² = 0.08, p < 0.0001). Given that behavioral change is the ultimate objective of gatekeeper education, this finding has strong practical relevance, supporting the utility of online formats for scalable implementation in resource-limited or remote settings.

### 3.3 Changes in attitudes toward suicide

Among the 10 subdomains assessed by the ATTS, both groups demonstrated improvements in accepting attitudes, prevention awareness, and readiness to intervene (d = 0.40–0.73; p < 0.01). The online group showed significantly greater reductions in inhibited attitudes (partial η² = 0.19, p < 0.0001), lack of understanding (η² = 0.08), and perceived motives for suicide (η² = 0.05), indicating its effectiveness in reshaping negative or stigmatizing beliefs.

**Figure 2:**
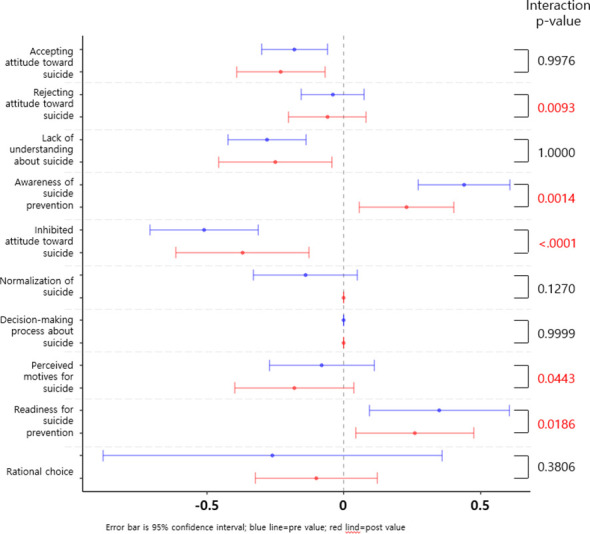
Pre-Post Evaluation of Attitudes Toward Suicide in the Online and Face-to-Face Gatekeeper Training Groups. Pre- (blue) and post-intervention (red) means with 95% confidence intervals are shown for each attitude subdomain. The x-axis indicates the change direction and magnitude. Interaction p-values reflect group x time effects (ANCOVA, adjusted for baseline). Significant between-group differences were observed in rejecting attitude, awareness, inhibited attitude, perceived motives, and readiness for prevention. See **Table 3** for detailed statistics.

While no significant group × time interactions were found for decision-making autonomy, normalization of suicide, or rational choice, modest within-group improvements were observed in both formats. These findings demonstrate that online education can influence not only knowledge and behavior but also complex affective and cognitive dimensions related to suicide prevention (**Table 3**, **Figure 2**). All relevant statistics, including means, standard deviations, t-test results, effect sizes, 95% confidence intervals, and interaction p-values are presented in **Tables 2** and **Table 3**. Effect plots are visualized in **Figures 1** and **2**.

**Table 3:** Pre–post evaluation of attitudes toward suicide.

Variable	Group	Pre	Post	Difference (=post-pre)†	p-value of group††
Accepting attitude toward suicide	Online	2.92±0.59	2.74±0.60	-0.18±0.44^**^	0.9976
	Face-to-Face	3.03±0.61	2.80±0.81	-0.23±0.57^**^	
Rejecting attitude toward suicide	Online	3.59±0.61	3.55±0.70	-0.04±0.42	0.0093
	Face-to-Face	3.23±0.72	3.17±0.72	-0.06±0.50	
Lack of understanding about suicide	Online	3.18±0.40	2.90±0.51	-0.28±0.52^***^	1.0000
	Face-to-Face	3.14±0.50	2.89±0.74	-0.25±0.73^*^	
Awareness of suicide prevention	Online	3.65±0.58	4.09±0.55	0.44±0.61^***^	0.0014
	Face-to-Face	4.09±0.55	4.32±0.57	0.23±0.61^*^	
Inhibited attitude toward suicide	Online	2.73±0.63	2.22±0.69	-0.51±0.72^***^	<.0001
	Face-to-Face	2.09±0.78	1.72±0.84	-0.37±0.86^**^	
Normalization of suicide	Online	3.16±0.59	3.02±0.66	-0.14±0.69	0.1270
	Face-to-Face	3.31±0.64	3.31±0.82	0.00±0.00	
Decision-making process about suicide	Online	2.88±0.51	2.88±0.54	0.00±0.00	0.9999
	Face-to-Face	3.10±0.57	3.10±0.60	0.00±0.00	
Perceived motives for suicide	Online	2.78±0.84	2.70±0.90	-0.08±0.70	0.0443
	Face-to-Face	2.48±0.67	2.30±0.67	-0.18±0.77	
Readiness for suicide prevention	Online	3.47±0.66	3.82±0.62	0.35±0.93^*^	0.0186
	Face-to-Face	3.81±0.62	4.07±0.85	0.26±0.76^*^	
Rational choice	Online	2.59±0.80	2.33±0.99	-0.26±2.26	0.3806
	Face-to-Face	2.30±0.83	2.20±1.06	-0.10±0.79	

The numerical variable is presented by mean±SD.

**†**Paired t-test between pre and post; *: <0.05, **: <0.01, ***: <0.0001.

**††**P-value derived from ANCOVA, adjusted for baseline scores.

## 4 Discussion

The present study demonstrates that both face-to-face and online formats of the Suicide CARE 2.0 gatekeeper training significantly enhanced suicide-related knowledge, attitudes, and behavioral intentions among mental health professionals. These findings align with previous studies reporting comparable efficacy between online and traditional gatekeeper training (6, 7). Our findings also contribute to the growing literature supporting the scalability of e-health interventions for suicide prevention (15, 16).

Importantly, gatekeeper training programs, particularly those designed for non-clinicians such as educators and community workers, have consistently been shown to improve proximal outcomes such as increased suicide-related knowledge, preparedness, and willingness to intervene (8–10). These outcomes are especially critical in community and non-medical settings, where early detection can prevent suicidal crises from escalating.

In the Korean context, the Suicide CARE program has been culturally tailored and widely implemented, showing long-term efficacy and adaptability to various populations, including adolescents and frontline workers (3–5). Our results further validate the program’s effectiveness even when delivered digitally, suggesting promising implications for broader dissemination, especially in remote or underserved regions (11, 12).

While several studies support the long-term impact of gatekeeper interventions on attitudes and behavioral intentions, findings regarding sustained change are mixed (9, 10). This highlights the need for booster training and periodic reinforcement, particularly for attitudinal components such as reducing suicide stigma and increasing empathy toward high-risk individuals. Institutional-level factors such as administrative support, organizational readiness, and policy mandates have also been identified as critical to the success and sustainability of suicide prevention programs (17, 18). Integrating gatekeeper training into institutional structures, such as schools, hospitals, and community centers, may improve long-term sustainability and alignment with national mental health strategies (20).

Despite its strengths, the online delivery format may face challenges related to learner engagement, emotional immersion, and real-time interaction. Future research should explore hybrid or augmented models that combine the scalability of digital platforms with the interpersonal depth of face-to-face learning (15, 16, 20). Technological enhancements, such as video-based narratives, peer-led discussion, and AI-assisted feedback mechanisms, may further improve engagement and knowledge retention.

A notable strength of this study is the use of validated measures to ensure reliable outcome assessment across multiple domains. Additionally, having the same experienced instructor deliver training across both groups minimized variability in content delivery and controlled for instructor-related bias. The high response rates for both pre- and post-intervention surveys further indicate good participant adherence and internal validity, even amid the constraints of the COVID-19 pandemic.

Nonetheless, several limitations should be acknowledged. First, the sample consisted of highly motivated volunteers, which may limit the generalizability of findings to broader or less-engaged populations. Second, the study did not include measures of subjective satisfaction, engagement levels, or learner preferences, factors that could inform future digital content optimization and instructional design. Third, the short-term follow-up precluded evaluation of long-term skill retention or translation into real-world gatekeeping behaviors. Lastly, while the sample size was adequate to detect main effects, the power may have been insufficient for subgroup analyses, particularly for more nuanced attitudinal shifts.

From a policy perspective, our findings support the incorporation of gatekeeper training into national suicide prevention strategies. Online delivery, in particular, presents a scalable, cost-effective modality well suited for resource-limited settings, rural regions, and public health emergencies, including the COVID-19 pandemic (19). To ensure quality and sustainability, digital dissemination should be supported by standardized curricula, instructor certification programs, and continuous fidelity monitoring. Learning management systems and mobile-based reinforcement tools may further enhance engagement and long-term retention. Additionally, implementing national tracking systems for trained gatekeepers could improve post-training support and reduce attrition in suicide prevention competencies over time (8).

In conclusion, this study adds to the growing evidence base for the effectiveness and scalability of online gatekeeper training in suicide prevention. While face-to-face instruction continues to offer distinct cognitive and interpersonal benefits, digital formats are emerging as equally effective and more flexible alternatives, mainly when based on structured, theory-driven models such as Suicide CARE.

## Data Availability

The datasets presented in this article are not readily available because. These data are not publicly available. Requests to access the datasets should be directed to Jong-Woo Paik, paikjw@khu.ac.kr.
